# Distinctiveness of symptoms of prolonged grief, depression, and post-traumatic stress in bereaved children and adolescents

**DOI:** 10.1007/s00787-012-0307-4

**Published:** 2012-07-12

**Authors:** Mariken Spuij, Ellen Reitz, Peter Prinzie, Yvonne Stikkelbroek, Carlijn de Roos, Paul A. Boelen

**Affiliations:** 1 Department of Child and Adolescent Studies, Utrecht University, Utrecht, The Netherlands; 2 Psychotrauma Centre for Children and Youth, Rivierduinen Mental Health Care, Leiden, The Netherlands; 3 Department of Clinical and Health Psychology, Utrecht University, PO Box 80140, 3508 TC Utrecht, The Netherlands

**Keywords:** Prolonged grief disorder, Complicated grief, Depression, Post-traumatic stress disorder, Children, Adolescents

## Abstract

Studies among adults have shown that symptoms of prolonged grief disorder (PGD) are distinct from those of bereavement-related depression and post-traumatic stress-disorder (PTSD). This study was an attempt to replicate this finding in two distinct samples of bereaved children (*N* = 197; aged 8–12 years) and adolescents (*N* = 135; 13–18 years), confronted with the death of a parent, sibling or other close relative. Using confirmatory factor analyses, we compared the fit of a one-factor model with the fit of a three-factor model in which symptoms formed three distinct, correlated factors. In both samples, findings showed that the model in which symptoms of PGD, depression, and PTSD loaded on separate factors was superior to a one-factor model and displayed excellent model fit. Summed scores on the PGD, depression, and PTSD items were significantly associated with functional impairment, attesting to the concurrent validity of the PGD, depression, and PTSD factors. The current findings complement prior evidence from adult samples that PGD is a distinct syndrome and suggest that PGD symptoms should be addressed in the assessment and treatment of bereaved children and adolescent seeking help following their loss.

## Introduction

There is growing evidence that the death of a loved one can precipitate the development of different forms of psychopathology, including depressive and anxious symptoms and syndromes, and prolonged grief disorder (PGD), previously named complicated grief [1–3]. PGD is a syndrome that includes persistent, disruptive yearning, trouble accepting the death, detachment, excessive bitterness, difficulties to move on, and a sense that the loss has shattered one’s view of self, life, and future, present to the point of impairment beyond 6 months post-loss [2–4].

Evidence of the existence and clinical significance of PGD has mainly been gathered in adult samples. Nonetheless, there is growing evidence that PGD symptoms can also occur in bereaved children and adolescents. For instance, in a study among 146 (11- to 23-year-old) friends and acquaintances of suicide victims, Melhem et al. [5] found that symptoms of what they called “traumatic grief” assessed as 6 months post-loss clustered together and predicted symptom-levels of depression and post-traumatic stress disorder (PTSD) at subsequent assessments. In another study among 129 parentally bereaved children (aged 7–18 years), PGD symptoms were associated with significant functional impairment beyond concurrent depression and PTSD [6].

Findings that PGD symptoms predict health impairments over and above other forms of distress (e.g., depression and PTSD) attest to the incremental validity of PGD. However, the phenomenology of dysfunctional grief in children and adolescents is still not very well understood. For instance, in contrast with the adult literature [1–3], few studies have examined the factorial distinctiveness of PGD in children and adolescents. Such research is important because it can enhance our knowledge of the generalizability of adult findings to younger bereaved individuals. This is timely given upcoming revisions of the Diagnostic and Statistical Manual of Mental Disorders (DSM) to create the fifth edition (DSM-5) that is likely to include a new disorder of grief [4, 7]. Moreover, research on the distinctiveness versus overlap of PGD, depression, and anxiety in bereaved children and adolescents can inform theorizing and research about underlying mechanisms of post-loss psychopathology and the development of methods for the assessment and treatment of such psychopathology.

There are only a few studies which have addressed the distinctiveness of PGD in children and adolescents. For instance, Melhem et al. [5] subjected items of the Texas Revised Inventory of Grief (TRIG; [8]) to a principal component analysis and found these items to cluster together into one cluster of traumatic grief and a second cluster of what they called “milder or even normal grief reactions” (p 1411). Dillen et al. [9] conducted two studies, one among 14- to 18-year-old adolescents confronted with the death of a grandparent, and a second among 12- to 19-year-old adolescents confronted with losses of different relatives. Both studies indicated that symptoms of PGD, depression, and anxiety clustered into three distinct factors. These studies provide preliminary support of the distinctiveness of PGD. Nonetheless, the study by Melhem et al. [5] is limited by its reliance on exploratory (and not confirmatory) factor analysis and the use of the TRIG that was developed as a measure of normal grief, not PGD [10]. The two studies by Dillen et al. [9] are limited by the fact that they only relied on children confronted with the loss of a grandparent (Study 1), did not examine the PGD distinctiveness in younger children, below 12 years of age (Study 2), and included no assessment of PTSD symptoms (Studies 1 and 2) which leaves the distinctiveness of PGD and PTSD still unexamined.

The aim of the current analyses was to address gaps in the research literature with regard to the phenomenology of bereavement-related emotional distress in children and adolescents. To this end, we examined the distinctiveness of symptoms of PGD, PTSD, and depression, in separate samples of 8- to 12-year-old children and 13- to 18-year-old adolescents, all confronted with the death of a parent, sibling, or other close loved one. Two related reasons guided our decision to conduct analyses in separate samples; the first is that the cognitive capacity to verbalize and contemplate the consequences of loss differs between children in primary versus secondary education [11], the second is that, accordingly, we used two different (though strongly similar) measurement instruments to assess PGD symptoms among 8- to 12-year-old children and 13- to 18-year-old adolescents, respectively [12] (see below).

Using confirmatory factor analysis (CFA), our first aim was to test the prediction that a three-factor model with symptoms loading on three distinct factors of PGD, depression, and PTSD, would be more suitable than a one-factor model. Our second aim was to examine associations between PGD, depression, and PTSD symptom-clusters on the one hand and indices of functional impairment on the other. We expected that the three symptom-clusters would all be correlated with functional impairment.

## Method

### Participants and procedure

Data were available from 332 children and adolescents, aged 8–18 years. Approximately, half of all participants were recruited via lay mental health care workers who organize individual support and mutual support groups for bereaved children and adolescents. Other participants were recruited via out-patient mental health care clinics in The Netherlands. Questionnaires administered for the present study were completed in the presence of either a research assistant or a health care worker. Assent was obtained from children (aged 8–12 years), and informed consent from parents and adolescents (aged 13–18 years). For the present study, separate analyses were conducted with data from *N* = 197 children (8–12 years) and *N* = 135 adolescents (13–18 years). Characteristics of the participants are shown in Table 1. Most participants had suffered the death of a parent, mostly due to an illness.

**Table 1:** Demographic variables, loss-related characteristics, and symptom-scores in children and adolescents samples

	Children (*N* = 197)	Adolescents (*N* = 135)
*Demographic characteristics*		
Gender, *N* (%)		
Boys	99 (50.3)	44 (32.6)
Girls	98 (49.7)	91 (67.4)
Age (years), *M* (SD); range	9.9 (1.3); 8–12	14.9 (1.5); 13–18
*Loss characteristics*		
Deceased is *N* (%)		
Mother	38 (19.3)	42 (31.1)
Father	123 (62.4)	63 (46.7)
Sibling	16 (8.1)	13 (9.6)
Other relative	17 (8.6)	15 (11.1)
Cause of death is *N* (%)		
Illness	105 (53.3)	77 (57.0)
Violent (accident, suicide, homicide)	48 (24.4)	26 (19.3)
Unexpected medical cause	34 (17.3)	28 (20.7)
Other cause	8 (4.1)	4 (3.0)
Experienced loss as unexpected? *N* (%)		
Yes	57 (28.9)	46 (34.1)
No	140 (71.1)	86 (63.7)
Time since loss (months) (*M* (*SD*); range)	31.7 (23.6); 1–119	34.0 (28.4); 2–119
Symptom Severity Scores		
Prolonged Grief (IPG)	50.8 (11.2)	50.3 (12.8)
Depression (CDI)	6.4 (6.6)	12.8 (7.5)
PTSD (CPSS)	13.3 (9.4)	14.5 (10.7)

Samples sizes differ due to occasional missing values

*CDI* Children’s Depression Inventory, *CPSS* Child Post-traumatic Stress-Disorder Symptom Scale, *IPG* Inventory of Prolonged Grief

* *p* < 0.05, ** *p* < 0.01, *** p < 0.001

### Measures

#### Demographic and loss-related variables

We registered the participant’s age and gender and collected information about the relationship to the deceased (categorized as mother, father, sibling, or other loved one), cause of death [loss was due to illness, a violent cause (accident, suicide, homicide), unexpected medical cause (e.g., heart attack), or some other cause], whether the death was experienced as unexpected (yes/no), and the time passed since the death occurred.

#### Inventory of Prolonged Grief for Children (IPG-C) and Inventory of Prolonged Grief for Adolescents (IPG-A)

PGD items were taken from the IPG-C and IPG-A. The IPG-C and IPG-A are both 30-item questionnaires, designed to assess symptoms of PGD among children and adolescents, respectively. Both measures are based on the Inventory of Complicated Grief designed to assess PGD among adults [13]. The IPG-C and IPG-A are very similar, although the wording of some of the items differs between the versions. Sample items include “I want to go to places that are related to him/her”, “I find it difficult to love other people since she/he died” (IPG-C), and “I seek out and feel attracted to places and things that are associated with him/her”, “I feel unable to love other people or feel distant from other people, since she/he died” (IPG-A). Respondents rated the frequency of each symptom in the preceding month, on 3-point scales (1 = almost never, 2 = sometimes, 3 = always). A recent study by Spuij et al. [12] supported the psychometric properties of both questionnaires. In the current study, the IPG-C yielded an α of 0.91 and the α of the IPG-A was 0.94.

#### Child PTSD Symptom Scale (CPSS)

Symptoms of PTSD were measures with the CPSS, a 24-item self-report questionnaire with 17 items corresponding to symptoms of PTSD as defined in DSM-IV [14], all rated on 4-point scales ranging from 0 (not at all/only once a week) to 3 (almost always/five or more times a week), and 7 items tapping functional impairment resulting from these symptoms, dichotomously rated as absent or present. The index event was defined as “the death of your loved one”. The CPSS was constructed by Foa et al. [15, 16] and has adequate psychometric properties. The α’s for the 17 symptoms and 7 functional impairment items in the children sample were 0.88 and 0.66, respectively. In the adolescent sample, the α’s were 0.92 and 0.67, respectively.

#### Children’s Depression Inventory (CDI)

Symptoms of depression were taken from the 27-item CDI [17, 18]. Each item contains three statements representing depressive symptoms at increasing levels of severity from which respondents select the statement that best describes their feelings in the preceding week, scored on a 0–2 scale, representing increasing symptom severity. In the children and adolescent sample, α’s were 0.85 and 0.87, respectively.

### Statistical analyses

Constraints of the sample sizes limited the number of items that could be included in the analyses. Consequently, we selected five items from each of the three symptom measures. Items for the factors were selected before any of the models were tested. Specifically, from the IPG-C and IPG-A, we selected the “yearning/longing” item, which is seen as a hallmark symptoms of PGD, and four items which are all included in the symptom criteria proposed by Prigerson et al. [2] and were among the items most highly correlated with the IPG-C and IPG-A total scores. For the PTSD factor, we selected two items from the reexperiencing cluster, two items from the avoidance cluster, and one item from the hyperarousal cluster that were most strongly correlated with the total score of each symptom-cluster. Finally from the CDI, we selected five items that represented DSM criteria for a depressive episode and were among the items most highly correlated with the CDI total score. Items are shown in Table 3.

To test the prediction that a three-factor model with symptoms loading on three distinct factors of PGD, depression, and PTSD would be more suitable than a one factor model, CFA was conducted using Mplus-6.12 [19]. As item scores were non-normally distributed, a robust weighted least squares (WLSMV) estimator was used. As missing data were rare (i.e., a maximum of 2 % per item) and completely at random, participants with missing data were included in the model estimations using full information maximum likelihood estimation [20]. Goodness-of-fit was evaluated using the comparative fit index (CFI), Tucker–Lewis index (TLI), and root mean square error of approximation (RMSEA). Although there is little consensus on cutoff values for adequate fit [21], conventional guidelines were followed whereby fit is considered adequate if CFI and TLI values are >0.90, and RMSEA is <0.05. χ^2^-Difference tests, calculated using the DIFF TEST option of Mplus-6.12 [19], were used to compare the fit of competing models. To examine associations between PGD, depression, and PTSD factor scores on the one hand, and CPSS functional impairment scores on the other hand, correlation and regression analyses were conducted.

## Results

### Descriptive statistics

Mean scores on the symptom measures of PGD, depression, and PTSD are shown in Table 1. Compared with reference groups for the IPG-C and IPG-A [12], the CDI [17, 18], and CPSS [15, 16], the current samples seemed best described as subclinical samples.

### Confirmatory factor analyses

Outcomes of the CFA among children showed that the three-factor model with symptoms loading on three distinct but correlated factors fit significantly better than the unitary model (∆χ^2^ = 43.708, ∆*df* = 3, *p* < 0.001). Fit estimates (shown in Table 2) showed that the three-factor model fits very well to the data as indexed by, for instance, a non-significant χ^2^ and very low RMSEA value. Correlations between factors were 0.54 for PGD with depression, 0.84 for PGD with PTSD, and 0.67 for depression with PTSD.

**Table 2:** Fit statistics for competing models of the factor structure of prolonged grief, depression, and post-traumatic stress symptoms

Samples and models	χ^2^	DF	*p*	χ^2^/*df*	TLI	CFI	RMSEA
Children							
One-factor model	140.941	90	0.0005	1.57	0.939	0.947	0.054
Three-factor model	75.355	87	0.8090	0.87	1.015	1.000	0.000
Adolescents							
One-factor model	132.298	90	0.0025	1.47	0.919	0.934	0.059
Three-factor model	94.412	87	0.2753	1.09	0.962	0.988	0.025

*CFI* comparative fit index, *RMSEA* root mean square error of approximation, *TLI* Tucker–Lewis index

Similarly, outcomes of the CFA among adolescents showed that the three-factor model fits significantly better than the unitary model (∆χ^2^ = 27.965, ∆*df* = 3, *p* < 0.001). Fit estimates (Table 2) showed that the three-factor model again fits very well to the data as indexed by, e.g., a non-significant χ^2^ and very low RMSEA value. Correlations between factors were 0.54 for PGD with depression, 0.80 for PGD with PTSD, and 0.66 for depression with PTSD.

Table 3 shows standardized factor-loadings from the three-factor models in both samples. All items loaded highly (>0.40) on their respective factors. Cronbach’s α’s of the items constituting each cluster were all ≥0.70, except α of the depression items in the children’s sample that was 0.58.

**Table 3:** Factor loadings for symptoms of prolonged grief, depression, and post-traumatic stress in children and adolescent samples

	Loadings on PG		Loadings on PTS factor		Loadings on depression factor	
	Children	Adolescents	Children	Adolescents	Children	Adolescents
Feels difficult to accept the loss	0.681	0.661				
Longing/yearning for lost person	0.654	0.627				
Feeling angry/bitter about the loss	0.776	0.806				
Life is only pleasant/meaningful with lost person	0.659	0.668				
Feeling like part of self died	0.588	0.571				
Intrusive thoughts about loss			0.772	0.737		
Experiencing flashbacks about loss			0.762	0.797		
Avoiding reminders of loss			0.567	0.595		
Restricted affect/numbness since loss			0.612	0.623		
Exaggerated startle since loss			0.661	0.591		
Reduced pleasure/fun doing things					0.701	0.717
Having thought about killing oneself					0.587	0.598
Feeling tired					0.449	0.465
Reduced pleasure/fun doing things					0.719	0.670
Having thought about killing oneself					0.538	0.585
Cronbach’s alpha of factor	0.70	0.75	0.74	0.75	0.58	0.700

*PG* prolonged grief, *PTS* post-traumatic stress

* *p* < 0.05, ** *p* < 0.01, *** *p* < 0.001

### Associations of PGD, depression, and PTSD factor scores with functional impairment

Items on the three factors were summed to obtain PGD, depression, and PTSD factor scores. In the children sample (*N* = 197), the functional impairment score from the CPSS was significantly correlated with the PGD factor score (*r* = 0.41), the depression factor score (*r* = 0.34), and the PTSD factor score (*r* = 0.46, *p*s < 0.001). When the three factor scores were simultaneously entered in a regression equation predicting CPSS functional impairment, a significant model emerged [*F*(3,194) = 22.70, *R* ^2^ = 26 %, *p* < 0.001], in which the PGD factor score (β = 0.19, *t* = 2.48, *p* < 0.05), the depression factor score (β = 0.15, *t* = 2.14, *p* < 0.05), and the PTSD factor score (β = 0.29, *t* = 3.63, *p* < 0.01), all explained unique variance in CPSS functional impairment.

In the adolescent sample (*N* = 135), the functional impairment score from the CPSS was also significantly correlated with the PGD (*r* = 0.42), depression (*r* = 0.44), and PTSD factor scores (*r* = 0.55, *p*s < 0.001). A regression analysis in which the CPSS impairment scores were regressed on factor scores entered together yielded a significant model [*F*(3,134) = 21.91, *R* ^2^ = 33 %, *p* < 0.001]. The PTSD factor score (β = 0.38, *t* = 4.03, *p* < 0.01), and, as a trend, the depression factor (β = 0.16, *t* = 1.79, *p* < 0.08), but not the PGD factor (β = 0.13, *t* = 1.50, *p* = 0.14) predicted unique variance in CPSS impairment scores.

### Associations of PGD, depression, and PTSD factor scores with background and loss-related variables

We examined if the PGD, depression, and PTSD factor scores varied a function of demographic variables (age, gender) and loss-related variables (kinship, cause of death, unexpectedness of death, and time since loss). In the children sample, no significant associations emerged, except for a negative correlation between the PTSD factor score and age (*r* = −0.23, *p* < 0.001). In the adolescent sample, age was positively correlated with PGD (*r* = 0.19), depression (*r* = 0.20), and PTSD (*r* = 0.17, *p*s < 0.05). In addition, girls had higher scores than boys on the PGD factor (*M* = 9.9, SD = 2.4 vs. *M* = 8.6, *SD* = 2.6; *F*(1,134) = 7.48, *p* < 0.01, *d* = 0.53), the depression factor (*M* = 3.1, SD = 1.9 vs. *M* = 1.7, SD = 1.8; *F*(1,134) = 16.16, *p* < 0.001, *d* = 0.76), and the PTSD factor (*M* = 3.9, SD = 3.2 vs. *M* = 2.6, SD = 2.8; *F*(1,134) = 5.49, *p* < 0.01, *d* = 0.43). No other significant associations emerged between factor scores and the loss-related variables in the adolescent sample.

## Discussion

The aim of the present study was to examine the distinctiveness and correlates of PGD, depression, and bereavement-related PTSD in bereaved children and adolescents. The main finding of this study was that, in both these groups, CFA showed that a model in which symptoms of PGD, depression, and PTSD loaded on three factors was superior to a unitary model and had excellent fit-estimates. Correlations between factors were moderate, indicating that the symptom-clusters represent distinguishable, but related constructs.

The current findings extend prior research in adult samples [1–3, 7] and children and adolescent samples [5, 9] which have shown that symptoms of intense yearning, difficulties to accept the loss, anger, and a sense that life is meaningless constitute a unique cluster of grief symptoms that can be distinguished from other emotional symptoms that can occur post-loss. The few prior studies among bereaved children that tested the distinctiveness of PGD were limited by their reliance on exploratory analyses, on children exposed to grandparental loss, relatively older children, and inclusion of generic measures of anxiety rather than PTSD [5, 9]. Thus, the present findings are an important extension of these prior findings by showing that the distinctiveness of PGD from its diagnostic “nearest neighbors” (i.e., syndromes from DSM which are phenomenologically close to PGD) is generalizable across a wide age range covering childhood and adolescence.

A second main finding was that, in both the children and adolescent samples, summed scores of the PGD, depression, and PTSD items were all significantly associated with a measure of functional impairment obtained from the CPSS. This attests to the concurrent validity of the three factors. In the children sample, the PGD, depression, and PTSD factor scores predicted unique variance in functional impairment when controlling for the shared variance between the three factor scores. In the adolescent sample, the PTSD factor was the single factor explaining unique variance in functional impairment. The findings could be taken to indicate that symptoms of PGD have a more specific invaliding impact in children compared to adolescents, and that symptoms of PTSD are relatively more invalidating in this later group. Yet, this conclusion seems premature, taking into account that our analyses relied on a single index of functional impairment, which was part of the PTSD measures, and that the size of the adolescent sample may have been too small to detect unique significant associations between PGD and functional impairment. Thus, future studies are needed to further examine this aspect of incremental validity of PGD in adolescents.

There are other limitations that should be taken into account. All data were gathered using self-report measures. It is possible that model fit and patterns of associations with other variables would have been different in case data were obtained from interview measures. A further limitation is that, given the variety of methods of recruitment, analyses relied on a rather heterogeneous group of children and adolescents. Thus, it would be interesting for future studies to further examine the factor structure and correlates of loss-related emotional symptoms following loss in more specified and homogeneous groups (e.g., children suffering severe emotional problems following loss). Finally, because of constraints of the sample size, we could only include a limited number of PGD symptom-criteria in our analyses. The distinctiveness of the more complete list of PGD symptoms thus requires further research with larger samples.

Notwithstanding these limitations, the results of this study provide further evidence that the complications of bereavement in children and adolescents may include symptoms of PGD that constitute a clinical entity that is distinct from bereavement-related depression and PTSD. These findings seem particularly timely given that a new disorder of grief will possibly be included in upcoming revisions of the DSM [4, 7, 22]. The results also underscore that different treatment methods may be required for the various syndromes that may develop in children and adolescents who fail to recover from a loss. For instance, activation-based methods may be indicated for depression, imaginary and in vivo exposure treatment may be useful for PTSD, and cognitive-behavioural interventions targeting dysfunctional meanings assigned to the loss and maladaptive coping may be indicated for typical grief-related symptoms. The development and evaluation of such specific treatment methods are important challenges for future research.
